# Growth performance and health of nursing lambs supplemented with inulin and *Lactobacillus casei*

**DOI:** 10.5713/ajas.18.0630

**Published:** 2019-02-07

**Authors:** Marco A Ayala-Monter, David Hernández-Sánchez, Sergio González-Muñoz, René Pinto-Ruiz, José A Martínez-Aispuro, Nicólas Torres-Salado, Jerónimo Herrera-Pérez, Adrian Gloria-Trujillo

**Affiliations:** 1 Program of Animal Science, Postgraduate College-Campus Montecillo, Texcoco, State of Mexico 56230, Mexico; 2 Faculty of Agricultural Sciences, Autonomous University of Chiapas, Chiapas 30470, Mexico; 3 Faculty of Veterinary Medicine No. 2, Autonomous University of Guerrero, Guerrero 41940, Mexico; 4 Department of Agricultural and Animal Production, Autonomous Metropolitan University Xochimilco, Mexico City 04970, Mexico

**Keywords:** Lambs, *Agave tequilana*, Inulin, Total Coliforms, Lactobacilli, Intestinal Health

## Abstract

**Objective:**

This experiment was designed to evaluate the effects of *Agave tequilana* inulin and *Lactobacillus casei* (*L. casei*) on growth performace, hematological variables, serum metabolites, and total coliforms in nursing lambs.

**Methods:**

The experimental design was completely randomized; treatments were T1, control (pre-starter concentrate, PC), T2: T1+2% inulin, and T3: T1+2% inulin+*L. casei*; treatments were compared with Tukey test (p≤0.05); and 45 new born Kathadin×Dorset lambs (4.8±0.8 kg birth weight) were the experimental units (15 per treatment). The variables were daily weight gain (DWG), dry matter intake and diarrheas incidence (%) during 56 d. Twenty-four hours after birth and at the end of the experiment, blood samples were collected to evaluate hematological variables and serum metabolites. Besides, the populations of total coliforms and lactobacilli were estimated in fecal samples.

**Results:**

Addition of agave inulin and *L. casei* increased (p≤0.05) DWG 356, 384, and 415 g/d, weaning weight 24.92, 26.18, and 28.07 kg, as well as lactobacilli population 5.79, 6.32, and 6.48 Log_10_ cfu/g, for T1, T2, and T3, respectively. Lambs fed *L. casei* had decreased (p≤0.05) populations of total coliforms (T1 = 6.18, T2 = 5.77, and T3 = 5.07 Log_10_ cfu/g), diarrheas incidence (T1 = 11.67%, T2 = 8.33%, and T3 = 5.0%), and serum cholesterol concentration (11% in T2 and 13% in T3, compared to control).

**Conclusion:**

The combination of *Agave tequilana* inulin and *L. casei* increases weight gain and improves intestinal health by reducing coliforms and diarrheas incidence in Katahdin× Dorset lambs during the pre-weaning period.

## INTRODUCTION

In intensive ovine production systems, the main objective is to obtain high percentages of weaned lambs to achieve larger quantities of meat and replacements. However, average mortality of neonate sheep is 15% [1] caused by inanition, exposure to low temperatures and deficient immunological protection. Regarding this last cause, transmission of antibodies in ruminants is blocked because of the histological nature of the placenta, and lambs are born without immunological protection [2]. Therefore, it is important that they ingest colostrum during the first hours of their lives [3] because later the passage of immunoglobulins through the intestinal villi is reduced and this limits the development of passive immunity [4]. Deficient immunological protection during lactation favors respiratory infections and diarrhea-related digestive infections [5] and lambs fed colostrum without specific antibodies might become sick [2].

Diarrhea in lambs is caused by etiological agents such as bacteria (*Escherichia coli*, *Salmonella* spp., *Clostridium* spp., *Campylobacter* spp.), parasites (*Cryptosporidium* spp. and *Giardia* spp.) and viruses (of the families *Adenoviridae*, *Coronaviridade*, *Rotaviriadae*). Therefore, diarrhea is a complex, multifactorial syndrome [5], which is economically important in the sheep industry because it causes low growth rates or death of lambs [6].

Prophylactic management of diarrheas is done through application of antimicrobials such as amino glucosides, sulfonamides, macrolides, tetracycline, and quinolones, but their continuous indiscriminate use causes bacteria to become resistant [7]. Probiotics and prebiotics are alternative supplements to antibiotics for controlling diarrheas [8,9].

Prebiotics, such as inulin, stimulate growth of probiotic bacteria [10], which act against pathogens by bacteriocin production and competitive exclusion [11]. This benefits intestinal microflora and lamb growth, while favoring the immune system [9]. In small ruminants, the use of inulin from chicory (*Cichorium intybus*) [12,13] is documented, but there are few investigations on nursing lambs [14]. In Mexico, inulin is obtained from species of the *Agavaceae* family [15], but no information was found of inulin used with lambs during nursing. We hypothesized that prebiotics and probiotics can improve lamb health and yield by modifying the microflora in the digestive tube. Thus, the objective of this study was to evaluate the effects of *Agave tequilana* inulin and *Lactobacillus casei* (*L. casei*) on growth performace, hematological variables, serum metabolites and total coliforms in nursing lambs

## MATERIALS AND METHODS

### Location

These study was conducted at the experimental farm of the Colegio de Postgraduados, Montecillo, Estado de Mexico (19° 27′ 38″ N, 98° 54′ 11′ W, 2250 masl), from May to August of 2016. The climate is temperate subhumid (Cwb), with summer rains and drought in winter (wo)(w), and moderate humidity (i′) g, with 663.7 mm average annual precipitation, and 15.8°C mean annual temperature. The lambs care and management procedures were conducted according to the guidelines established by the Animal Welfare Committee of the Colegio de Postgraduados.

### Animals, management and treatments

Forty-five new-born male Katadin×Dorset lambs (4.8±0.8 kg weight at birth) were used in the experiment. The lambs were obtained from a lot of second-partum ewes. After birth, the lambs were fed colostrum (Table 1). The umbilical cord of each lamb was disinfected (Negasunt, Bayer, Mexico City, Mexico) and lambs were housed with their mothers in individual pens equipped with feeder and waterer. When the ewes were fed, the lambs were kept in the creep feeding in the same pen. The treatments were randomly assigned to one of three treatments (T): T1, control (pre-starter concentrate, PC); T2, T1+2% inulin; and T3, T1+2% inulin+*L. casei* (n = 15, Table 2). The lambs remained with the ewes until weaning (60±2 d) under the same conditions. During lactation, the lambs were offered, besides nursing, pre-starter feed (Nulamb, Group Nutec, El Marques, Queretaro, Mexico; Table 3) and water, both *ad-libitum*.

The probiotic and inulin were administered orally with a syringe and oral cannula at 09:00 h during the 56 d (inulin was resuspended in distilled water). The probiotic and probiotic doses were adjusted in each evaluation periods according to the lambs live weight (Table 2). The prebiotic evaluated was Inulin Powder, Bestground (Jalisco, Mexico) and the probiotic was commercial Yakult (Yakult, Ixtapaluca, State of Mexico, Mexico), which contained 10^8^ cfu/mL of *L. casei*.

The diet provided to each ewe was 800 g/d of commercial concentrate (Ovina Reproductores, Agribrands Purina, Cuautitlan, State of Mexico, Mexico, 14% of crude protein [CP]), 1,200 g/d of oat hay (9.2% of CP), and 800 g/d of alfalfa hay (16.9% of CP, only during the first month of nursing) offered once a day at 08:00 h, and water *ad-libitum*.

### Analysis of colostrum, milk and pre-starter concentrate

Samples of colostrum and milk (50 mL) were collected by manual milking into plastic receptacles 6 h postpartum, on days 5 and 28 of nursing to determine the chemical characteristics (Table 1) with an ultrasonic analyzer (Lactoscan LA, Milkotronic, Nova Zagora, Bulgaria). In the colostrum samples, specific gravity was also measured to estimate the concentration of immunoglobulins [16] with a digital refractometer (Hanna 96801, Eibar, Spain). The chemical composition of the feed (Table 3) was determined according to AOAC methodology [17]: dry matter (DM; method 930.15), CP (method 984.13), ash (method 942.05) and ether extract (method 954.02). Neutral detergent fiber and acid detergent fiber was determined with the analyzer ANKOM (Ankom Technology Corp. A200 Fairport, NY, USA).

### Animal performance

Lamb weight was recorded at birth (BW, 6 h after birth). Daily weight gain (DWG, g/d) was estimated by weighing each lamb at birth and every 14 d (08:00 h). Weight at weaning (WW) was obtained on day 60±2. Total weight gain (kg) was calculated as the difference between WW and BW. Feed intake (g/d) was recorded every day, and estimated by group, calculating the difference between offered and rejected feed and dividing by the number of lambs in the group (n = 15).

### Hematological variables

Two blood samples, 24 h postpartum and at the end of the experiment (pre-prandial, 08:00 h), were collected (5 mL/lamb) by venopunction of the jugular vein. The first sample was collected in tubes with anticoagulant (BD Vacutainer, Cuautitlan, State of Mexico, Mexico; K2 ethylenediaminetetraacetic acid) for hematological evaluations, and the second was deposited in tubes without anticoagulant (BD Vacutainer, Mexico; Serum) for separating the serum. Both samples were placed immediately in refrigeration (4°C) until analysis. Determination or erythrocytes, hematocrit, hemoglobin and leucocyte differential was carried out in an automatized hematological analyzer (Sysmex XS-1000i, Kobe, Japan).

### Serum metabolites

The blood samples without anticoagulant were centrifuged (Sigma, 2-16KL, Osterode am Harz, Germany) at 3,500×g for 20 min. The obtained serum was conserved in Eppendorf tubes at −20°C until analysis. In each sample the serum concentrations of total cholesterol (TC, oxidase-peroxidase enzyme method), glucose (GLU, enzyme method), total protein (TP, Biuret method), albumin (ALB, Bromo cresol green method) were determined with specific kits acquired from Spinreact (Barcelona, Spain). Readings were done in a visible UV light spectrophotometer (Cary 1-E Varian, Mountain View, CA, USA) at 505, 540, and 630 nm wavelengths for TC and GLU, TP and ALB, respectively. The serum concentration of globulins was estimated by difference between total protein and albumin contents [18].

### Total coliforms and lactobacilli in feces (cfu/g)

On the last day of the experiment (09:00 h) fecal samples were collected (10 g/lamb) in sterile collection tubes by rectal stimulation and using latex gloves. The tubes were maintained at 4°C during transport to the laboratory. One gram of each sample was diluted in a Labcon tube with 9 mL peptonized solution (8.5%). It was then homogenized in vortex for serial dilutions of 10^−1^ to 10^−12^. Each dilution was seeded in triplicate 100 μL by the striate method in Petri dishes containing selective medium for coliforms (Mac Conkey, Sigma-Aldrich, Munich, Germany) or for lactic acid bacteria (de Man, Rogosa and Sharpe, Sigma-Aldrich, Germany) and incubated 24 and 48 h at 37°C, respectively. After incubation, colony-forming units were counted to estimate the microbial population, and the data were expressed with the base 10 logarithm function (log_10_). The material and the culture media used in this phase were sterilized 15 min in autoclave (Lab-Med, LMV40, Mexico City, Mexico) at 121°C and 15 psi, and the dilutions and bacterial seeding were performed in a laminar flow hood (Labconco, Logic A2 800, Kansas City, MO, USA).

### Diarrhea incidence

Diarrhea incidence was recorded for each lamb during the experiment (08:00 h), based on the presence of stains on the hind legs and accumulation of feces around the perianal area [12]. Lamb health was also monitored.

### Statistical analysis

The experimental design was completely randomized with 15 replications per treatment. The results were analyzed with PROC general linear model of SAS [19] and treatments were compared with the Tukey test (p≤0.05). The incidence of diarrheas was analyzed with the *X*^2^ test and microorganism count was expressed with the function log_10_.

## RESULTS AND DISCUSSION

### Lambs performance

Lambs birth weight was similar (p>0.05) between treatments, and average weight (4.8 kg) at birth (Table 4) was within the optimal range of 3.5 to 5.5 kg [1]. The DWG and weaning weight of lambs that received agave inulin or the combination of inulin with *L. casei* were 16% higher (p≤0.05) than those of the control group (Table 4). However, weight increase did not change (p>0.05) between lambs supplemented with inulin or with both inulin and *L. casei*. In addition, dry matter intake (DMI) did not differ (p>0.05) among treatments (Table 4).

Weight gain of lambs at weaning is due in part to nursing and the availability of feed *ad-libitum*, but the weight gain is related to digestion efficiency which is affected by the presence of prebiotics and probiotics [8]. During nursing, all lambs received colostrum and milk with similar chemical composition (Table 1), since the sheep were kept in analogous conditions of feeding, number and type of birth. The available pre-starter feed covered the nutrient requirements of lambs when the ewes’ milk production diminished. Nevertheless, it is important to underline that natural nursing improves lamb weight gains and immunological variables, as observed in our study [20]. Moreover, the use of lactic acid bacteria during nursing stimulates microbial growth and use of nutrients in the rumen [9]. Thus, the intake of solid diets and physiological development of the rumen is accelerated [21], shortening time to weaning and favoring productive response.

Better daily weight gain was also observed when mixtures of probiotic strains or inulin with *Enterococcus faecium* (*E. faecium*) were included in the diet of lambs during lactation [14,22], and the positive effects in DWG was also documented with probiotics in diets for growing lambs [23–25] or goat kids [26]. However, other studies do not report improved on DWG in lactating lambs [27], goat kids [12], or growing lambs [13] supplemented with probiotics and inulin. An increment in DWG is related to increased DMI [28], but in our study, feed intake did not change (p>0.05; Table 4) because of inulin and *L. casei* supplementation. Kazemi-Bonchenari et al [13] reported similar results when they fed a mixture of inulin and *E. faecium* for growing lambs. Moarrab et al [14], however, found that DMI tended to decrease in lambs receiving high doses of prebiotics and probiotics.

The DWG increasing (p≤0.05) without change (p>0.05) in DMI when inulin and *L. casei* were supplemented together could be related to an improvement in diet digestibility and feed efficiency as reported in other studies [13,14,23]. In addition, it has been shown that a favorable environmental condition for gastrointestinal microbes caused improvement in nutrients digestibility, and probiotic and prebiotic are shown to prepare these conditions in animal [10,11]. Probiotics and prebiotics have the potential to increase short chain fatty acids production and high ruminal levels of these metabolites improve nutrient digestibility due to their effect on bacteria growth and activity [29]. These symbiotic effects of inulin and *L. casei* also might explain the best DWG being observed when inulin and *L. casei* were combined in diet, leading to better feed efficiency, therefore DMI did not change between treatments.

### Hematological variables

Hematological variables in lambs supplemented with inulin and *L. casei* were not different (p>0.05) among treatments (Table 5), and the mean values are within the physiological interval reported in the literature for sheep [18]. The hematological variables reported in the literature regarding supplements of inulin and probiotic strains are not conclusive. Thus, no changes were found for growing goat kids [12] and lambs [13], whereas Hossein-Ali et al [24] and Hussein [30] reported increases in hemoglobin, hematocrit and erythrocytes in lambs fed probiotics. Moreover, El-Mehanna et al [25] and Bularon and Plata [26] observed increased leucocyte counts when probiotics were used with lambs and kids during lactation. The hematological variables are affected by age, environmental factors during sample processing, and characteristics and handling of lambs [31], although lamb health was good during our study.

### Serum metabolites

Mean values of TC, glucose, total protein, albumin and globulins in lamb serum were not different (p>0.05) among treatments, but at weaning the serum concentration of cholesterol decreased (p≤0.05) 11.6% in lambs that received inulin and 13.3% in lambs fed both inulin and *L. casei* (Table 6). The values of serum metabolites found in our study are within the normal range described for nursing lambs [18]. The absence of effects on serum concentration of glucose, total protein, albumin and globulins is consistent with reports for nursing lambs [14,25,28] and finishing lambs [13,26] supplemented with prebiotics and probiotics. In contrast, Abdel-Salam et al [23] and Hussein [30] report an increase in concentrations of total protein, albumin and globulins in lambs fed symbiotics and probiotics, respectively. However, according to Hossein-Ali et al [24], there is a decrease in concentrations of these metabolites in lambs fed probiotics. The differences in serum concentrations of the metabolites reported in the literature and those obtained in our study may be due to nutritional factors, type of prebiotic or probiotic (dose, time of administration), and breed, sex, age and state of health of the lambs.

At weaning, the concentration of serum cholesterol decreased (p≤0.05), congruent with Moarrab et al [14] and Saleem et al [27], who included inulin and probiotic strains in lamb diets during weaning. Reduction in the concentration of this metabolite is attributed to the hypocholesterolemic effect of inulin and deconjugation of bile fatty acids due to bile salt hydrolase activity produced by lactic acid bacteria such as *Lactobacillus* and *Bifidobacterium*. Consequently, the fatty acids are less soluble, their absorption in the intestine decreases, and blood concentration is reduced [32]. Because of the histological nature of the placenta in ruminants, lambs are born hypogammaglobulinemic [2,3], and they need to ingest colostrum, 180 to 210 mL/kg liveweight or 10% of their liveweight, for the first 4 h postpartum to receive passive immunity, since absorption decreases as of 6 h postpartum [1,4]. Transfer of passive immunity can be evaluated by measuring the serum concentration of total protein; levels below 5 d/dL are associated with hypogammaglobulinemia [17]. The serum concentration of total protein found in our study (Table 6) indicates that the colostrum ingested by the lambs during the first hours of life gave them passive immunity, as well as contributing growth factors and nutrients such as fats, lactose, vitamins and minerals [3].

### Total coliforms and lactobacilli in feces

Addition of inulin and *L. casei* (T3) decreased (p≤0.05) the population of total coliforms and increased (p≤0.05) that of lactobacilli in lamb feces, relative to the control group (Table 7). Our results are similar to those of Moarrab et al [14], who combined prebiotics and probiotics. Besides, Reddy et al [22] reported a decrease in fecal coliform count in lambs supplemented with probiotic strains of *Pediococcus acidiactici* and *Saccharomyces boulardii* during nursing. Before birth, the lamb digestive tube is sterile, after that it is colonized by microorganisms from the birthing channel and the environment. At that moment, establishment of ruminal and intestinal flora begins [21], and the contribution of prebiotics and probiotics is important. The results of our study show that lambs supplemented with inulin and *L. casei* together had lower total coliform populations, which could be related to the effect of lactic acid bacteria when they produce bacteriocins, hydrogen peroxide and lower pH, causing competitive exclusion of enteropathogens [11]. Prebiotics, such as inulin stimulate growth and activity of lactic acid bacteria, particularly of the genera *Lactobacillus* and *Bifidobacterium* [10], which have a positive effect on the microflora that maintains the intestinal epithelium healthy and promotes greater availability and absorption of nutrients through intestinal villi [14]. Congruent with this, the combination of inulin and *L. casei* in our study produced a synergic effect by reducing coliforms population and increasing total lactobacilli.

### Diarrhea incidence

In the control group, 11.67% of the lambs had diarrhea, whereas 5.0% of the lambs supplemented with inulin and *L. casei* had diarrhea during the second and third period of evaluation (Table 8). This is associated with the transition from pre-ruminant to ruminant [21], during which the basic feed of the lambs is their mothers’ milk. However, when ewe milk production diminishes as lactation progresses, the lambs increase intake of solid feed to satisfy their nutritional requirements. This can alter the intestinal flora and lead to mechanical diarrheas. Therefore, it is important that the lambs receive solid feed at an early age so that the ruminal flora can establish and adapt [21,33], whereby decreasing the stress caused by abrupt change in feeding. Otherwise, the glucocorticoid hormones released due to stress will decrease DMI, lambs will have low growth rate [34] and susceptibility to diarrhea-causing pathogens will increase.

The positive response to prebiotics and probiotics could be related to stimulation of the immune system, decrease in stress, and prevention of diarrheas, which optimizes growth of healthy lambs, although the results can vary due to feeding practices, handling, and age at weaning [20,28]. However, the low incidence of diarrhea in our study was due to the combined effect of probiotic and prebiotic, which can produce favorable environmental condition and growth factors for gastrointestinal benefits bacteria [10,11,29] and led to the increase of tottal lactobacilli (Table 7). In this way, *L. casei* and acido lactic bacteria reduced total coliforms through competitive exclusion. In addition, results can be associated with the prebiotic effect of inulin on caliciform cells that produce mucus, which forms a viscous layer on the intestinal epithelial lining and limits adhesion of enteropathogens. They also modulate the composition of intestinal flora by increasing the population of lactobacilli and stimulate the immune system [9,10], and reduce diarrheas.

## CONCLUSION

Supplementation with the combination of *Agave tequilana* inulin and *L. casei* increases weight gain and improves intestinal health by reducing coliforms and diarrheas incidence in Katahdin×Dorset lambs during the pre-weaning period.

## Figures and Tables

**Table 1:** Chemical composition of colostrum and milk (average±standard deviation)

Composition (%)	Colostrum (6 h)	Milk	

		Early lactation (5 d pospartum)	Mid lactation (28 d pospartum)
Fat	8.9±3.3	5.8±2.4	5.2±1.4
Protein	7.5±0.4	4.1±1.4	3.7±0.7
Lactose	3.5±0.3	6.2±2.1	5.5±1.0
Solid not fat	16.0±4.3	11.3±3.9	10.0±1.8
Density	1,056±21.7	1,031±9.7	1,032±2.6

**Table 2:** Experimental treatments

Periods (d)	Control (C)1)	C+2% inulin2) g4)	C+2% inulin+*Lactobacillus casei*3) g and mL5)
1–14	-	5	5 and 15
15–28	-	8	8 and 30
19–42	-	12	12 and 45
43–56	-	15	15 and 45

1) Pre-starter concentrate Nulamb (Group Nutec, El Marques, Queretaro, Mexico).

2) Inulin Powder (Bestground, Jalisco, Mexico) contributed 2% based on the consumption of dry matter and was based on the lanbs LW.

3) Yakult (Yakult, Ixtapaluca, State of Mexico, Mexico; 10^8^ cfu/mL).

4) Grams of inulin per lamb per day.

5) Grams of inulin and milliliters of Yakult per lamb per day.

**Table 3:** Chemical composition of pre-starter concentrate1)

Item	Dry matter basis (%)
Dry matter	92.51
Crude protein	18.47
Ether extract	3.50
Ash	6.71
Neutral detergent fiber	24.74
Acid detergent fiber	6.21

1) Pre-starter concentrate Nulamb (Group Nutec, El Marques, Queretaro, Mexico).

**Table 4:** Growth performance of nursing lambs with an inulin supplement of Agave and *Lactobacillus casei*

Item	Control (C)1)	C+2% inulin2)	C+2% inulin+*L. casei*3)	SEM	p-value
Number of lambs	15	15	15	-	-
Birth weight (kg)	4.97	4.65	4.83	0.21	0.58
Weaning weight (kg)	24.92^b^	26.18^ab^	28.07^a^	0.73	0.01
Total weight gain (kg)	19.96^b^	21.52^ab^	23.24^a^	0.61	0.002
Average daily gain (g/d)	356^b^	384^ab^	415^a^	0.01	0.002
Dry matter intake (g/d)	250	252	247	-	-

SEM, standar error of the mean.

1) Pre-starter concentrate Nulamb (Group Nutec, El Marques, Queretaro, Mexico).

2) Inulin Powder (Bestground, Jalisco, Mexico).

3) Yakult (Yakult, Ixtapaluca, State of Mexico, Mexico; 10^8^ cfu/mL).

a,b Values with different literal in a row indicate differences (p<0.05).

**Table 5:** Hematological variables of nursing lambs with an inulin supplement of Agave and *Lactobacillus casei*

Item	Control (C)1)	C+2% inulin2)	C+2% inulin+*L. casei*3)	SEM	p-value
Erythrocytes (10^6^/mL)	5.06	4.91	4.92	0.16	0.76
Hemoglobin (g/dL)	12.95	11.93	13.64	0.62	0.16
Hematocrit (%)	25.65	24.51	24.53	0.71	0.43
MCV (fL)	50.71	50.00	49.94	0.31	0.17
MCH (pg)	49.93	48.33	50.53	1.83	0.68
MCHC (g/dL)	25.33	26.92	25.23	1.00	0.42
Platelets (mil/μL)	260.33	349.75	285.08	27.14	0.06
Leukocytes (10^3^/mL)	46.39	51.97	51.99	9.63	0.89
Lymphocytes (%)	70.77	71.26	62.01	3.99	0.19
Monocytes (%)	5.30	4.86	3.16	0.74	0.11
Segmented (%)	18.02	14.32	19.83	3.53	0.53
Basophils (%)	1.34	1.40	1.32	0.05	0.53
Eosinophils (%)	0.80	1.13	1.62	0.26	0.10

SEM, standar error of the mean; MCV, medium corpuscular volume; MCH, medium corpuscular hemoglobin; MCHC, medium corpuscular hemoglobin concentration.

1) Pre-starter concentrate Nulamb (Group Nutec, El Marques, Queretaro, Mexico).

2) Inulin Powder (Bestground, Jalisco, Mexico).

3) Yakult (Yakult, Ixtapaluca, State of Mexico, Mexico; 10^8^ cfu/mL).

**Table 6:** Blood metabolites of nursing lambs with an inulin supplement of Agave and *Lactobacillus casei*

Item	Control (C)1)	C+2% inulin2)	C+2% inulin+*L. casei*3)	SEM	p-value
Cholesterol (mg/dL)					
Post-birth (24 h)	105.32	98.75	96.58	6.54	0.62
Weaning (60±2 d)	79.33^a^	70.07^ab^	68.76^b^	2.70	0.01
Glucose (mg/dL)					
Post-birth (24 h)	86.35	83.82	91.80	3.45	0.25
Weaning (60±2 d)	71.75	73.90	75.26	1.05	0.07
Total protein (g/dL)					
Post-birth (24 h)	6.96	7.10	6.71	0.25	0.54
Weaning (60±2 d)	6.65	7.06	6.84	0.15	0.19
Albumin (g/dL)					
Post-birth (24 h)	2.53	2.51	2.68	0.91	0.34
Weaning (60±2 d)	3.24	3.16	3.08	0.06	0.25
Globulin (g/dL)					
Post-birth (24 h)	4.42	4.58	4.02	0.24	0.26
Weaning (60±2 d)	3.40	3.90	3.75	0.15	0.07

SEM, standar error of the mean.

1) Pre-starter concentrate Nulamb (Group Nutec, El Marques, Queretaro, Mexico).

2) Inulin Powder (Bestground, Jalisco, Mexico).

3) Yakult (Yakult, Ixtapaluca, State of Mexico, Mexico; 10^8^ cfu/mL).

a,b Values with different literal in a row indicate differences (p≤0.05).

**Table 7:** Total coliforms and lactobacilli in feces (Log10 cfu/g) of nursing lambs with an inulin supplement of Agave and *Lactobacillus casei*

Item	Control (C)1)	C+2% inulin2)	C+2% inulin+*L. casei*3)	SEM	p-value
Total coliforms	6.18^a^	5.77^a^	5.07^b^	0.12	0.0001
Lactic acid bacteria	5.79^b^	6.32^ab^	6.48^a^	0.16	0.02

SEM, standar error of the mean.

1) Pre-starter concentrate Nulamb (Group Nutec, El Marques, Queretaro, Mexico).

2) Inulin Powder (Bestground, Jalisco, Mexico).

3) Yakult (Yakult, Ixtapaluca, State of Mexico, Mexico; 10^8^ cfu/mL).

a,b Values with different literal in a row indicate differences (p≤0.05).

**Table 8:** Diarrhea incidence (%) of nursing lambs with an inulin supplement of Agave and *Lactobacillus casei*

Item	Control (C)1)	C+2% inulin2)	C+2% inulin+*L. casei*3)	*X*^2^ 4)	p-value
Normal feces	88.33	91.67	95.00	1.7455	0.4178
Diarrheas	11.67	8.33	5.00		

1) Pre-starter concentrate Nulamb (Group Nutec, El Marques, Queretaro, Mexico).

2) Inulin Powder (Bestground, Jalisco, Mexico).

3) Yakult (Yakult, Ixtapaluca, State of Mexico, Mexico; 10^8^ cfu/mL).

4) Value of the Chi square test.
